# Socioeconomic and genomic roots of verbal ability from current evidence

**DOI:** 10.1038/s41539-022-00137-8

**Published:** 2022-09-09

**Authors:** Guang Guo, Meng-Jung Lin, Kathleen Mullan Harris

**Affiliations:** 1 Department of Sociology, University of North Carolina at Chapel Hill, Chapel Hill, NC USA; 2 Carolina Population Center, University of North Carolina at Chapel Hill, Chapel Hill, NC USA

**Keywords:** Interdisciplinary studies, Education, Sociology

## Abstract

This research examines how the human genome and SES jointly and interactively shape verbal ability among youth in the U.S. The youth are aged 12–18 when the study starts. The research draws on findings from the latest GWAS as well as a rich set of longitudinal SES measures at individual, family and neighborhood levels from Add Health (*N* = 7194). Both SES and genome measures predict verbal ability well separately and jointly. More interestingly, the inclusion of both sets of predictors in the same model corrects for about 20% upward bias in the effect of the education PGS, and implies that about 20–30% of the effects of parental SES are not environmental, but parentally genomic. The three incremental *R*^*2*^s that measure the relative contributions of the two PGSs, the genomic component in parental SES, and the environmental component in parental SES are estimated to be about 1.5%, 1.5%, and 7.8%, respectively. The total environmental *R*^*2*^ and the total genomic *R*^*2*^ are, thus, 7.8% and 3%, respectively. These findings confirm the importance of SES environment and also pose challenges to traditional social-science research. Not only does an individual’s genome have an important direct influence on verbal ability, parental genomes also influence verbal ability through parental SES. The decades-long blueprint of including SES in a model and interpreting their effects as those of SES needs to be amended accordingly. A straightforward solution is to routinely collect DNA data for large social-science studies granted that the primary purpose is to understand social and environmental influences.

## Introduction

Cognitive ability has been shown to be one of the most important predictors of life outcomes such as educational attainment, occupational achievement, income, wealth, and health^1–6^. For decades, tests of cognitive ability and related achievement tests were routinely and nearly universally used in elementary and secondary education, college admissions, and admissions of graduate schools and professional schools in the United States^7,8^.

A critical question about cognitive ability is how much of it is innate and how much is environmental. The answer is related to how much cognitive ability could be improved by schooling and other individual and public efforts. Past few decades have seen two major advances in the understanding of genetic influences on cognitive ability. Behavior geneticists, taking advantage of genetic relatedness among blood relatives, have long established a substantial genetic base for cognitive ability, reporting a heritability estimate of 50% or more from adolescence through older age^9–12^. The second major advance came with the genome-wide association study (GWAS) of educational attainment^13^ and cognitive ability^14^. These GWAS yield a large number of genetic variables at molecular level that predict cognitive ability.

In this article, we report findings from research that investigates how the human genome and socioeconomic (SES) environment jointly and interactively shape verbal ability among youth in the United States. The research draws on results from two recent GWAS as well as a rich set of longitudinal SES measures at individual, family and neighborhood levels from the National Longitudinal Study of Adolescent to Adult Health (Add Health)^15^.

Our work promises significant improvements in the assessment of the relative importance of SES and genomic factors for verbal ability. First, recent GWAS provide an opportunity for genomic influence to be measured at individual DNA level. Twin studies decompose the total variance of cognitive ability into proportions due to genetic, shared environmental and unshared environmental factors at population level. Traditional twin data whose genetic information is based on genetic relatedness alone without DNA measures cannot investigate the relative contributions from genetics and environment as do data with correlated genetic and SES measures at the individual level. Second, we build our analysis from a social-science model of verbal ability; such a model often includes a rich set of longitudinal measures of SES. This approach allows us to examine the changes in a traditional social-science model of verbal ability after adding genomic measures. In an era when DNA evidence for human traits was by and large unavailable, twin studies understandably focused on genetic evidence. Typically, environmental information was insufficiently addressed in twin analysis. Understanding the relative importance of genomics and SES requires both sets of measures at individual level. Third, simultaneous inclusion of offspring genomic and family-SES data allows us to evaluate the genomic component within family SES^16,17^, which in turn allows us to correct for substantial upward bias in the effects of offspring genome and parental SES. Our findings have important implications and challenges for researchers dedicated to establishing genetic impact on cognitive ability and for researchers with a decades-long tradition of examining social and environmental impact on cognitive ability.

The influence of family SES on cognitive measures has been investigated extensively in a variety of academic fields over the past few decades. These previous analyses suggest the kinds of SES measures and analysis models that we can use in this research. SES is an umbrella concept with multiple dimensions at individual, family and community levels that attempts to capture family financial resources, knowledge, social connections, and the larger social context. SES is typically measured by parental income, education and occupation, one vs. two biological parents in the household, sibship size, quality of neighborhood, and quality of school^18–21^.

SES is most frequently measured by family income or poverty^21–23^. Other family characteristics such as family instability^24^ and parenting style^25^ are also examined. Entwisle and Alexander^26^ concludes that family SES has a larger impact than the interruption of schooling from summer breaks on cognitive test scores in elementary schools. Using data from the Child Development Supplement in the Panel Study of Income Dynamics, Hill^21^ shows that after income level is controlled, an increasing 5-year trend in income-to-needs income is positively associated with child achievement. In a meta-analysis of adoption studies of IQ, Locurto^27^ shows that adoption into high SES families raises the adopted children’s IQ by 10–12 points. These adoptive children tend to come from low SES families.

Mechanism studies explain why SES makes a difference in results of cognitive tests. Employing four waves of Dutch data, Kloosterman et al.^28^ shows that part of the relationship between parental education and children’s academic performance can be explained by parental reading and school involvement. Guo and Harris^29^ investigates the mechanisms through which family SES affects children’s cognitive ability. Using data from the National Longitudinal Survey of Youth (NLSY), they show that the effect of family SES is completely mediated by the intervening mechanisms measured by the latent factors of cognitive stimulation in home, home physical environment, parenting style, and child health at birth. Cognitive ability in the study is measured by four Peabody tests including a reading recognition test, a reading comprehension test, a mathematics assessment test, and a Peabody Picture Vocabulary test.

Hart and Risley^30^ observes children in 42 families for one hour per week for two and a half years and their calculation suggests that large differences exist in the total number of words heard by children from birth to age four across professional families (45 millions words), working class families (25 millions), and families in poverty (13 millions). Using the children of the NLSY, Farkas and Beron^31^ shows that by the 36th month of age, large gaps in vocabulary already emerge across social classes and racial/ethnic groups, and the gaps are not closed afterwards.

Formal schooling is commonly viewed as the most critical for the development of cognitive ability. Tests of cognitive ability are hardly meaningful out of the context of modern education. Dutch children’s schooling was delayed by the Nazi regime during WWII and these children’s IQ are seven points lower on average than those who went to school at normal ages after the war^32,33^ p. 41. In a longitudinal study of the effects of family SES, racial mix, and summer breaks on children’s mathematics achievement, Entwisle and Alexander^26^ concludes that “when school is in session, poor children and better-off children perform at almost the same level. Schools seem to be doing a better job than they have been given credit for (pp.82.)” They demonstrate the effect of schooling by showing the loss in mathematics scores after every summer break on the part of children in poverty relative to children not in poverty. Through a natural experiment, Cahan and Cohen^34^ reports a schooling effect distinct from the effect of biological age on an IQ test score. The study compares fourth graders and fifth graders (ages 9–11) who were essentially of the same age. Winship and Korenman^35^ reviews the literature that estimates the effect of education on cognitive ability and carefully reanalyzes the data from the NLSY used by Herrnstein and Murray^36^. They conclude that each year of education increases 2.7 points of IQ units. In this analysis, we consider respondents’ own schooling a paramount SES determinant of cognitive ability.

The afore-reviewed SES studies almost always assume explicitly or implicitly that all of the SES influences under consideration are environmental. These studies generally do not consider genomic influences. Nor do they address the issue that family SES itself could be partially genetic.

Getting to the root of cognitive ability requires DNA data. When social scientists reflect about genetic influences on cognitive ability, the first thing that often comes to mind is the well-publicized position that considers cognitive ability an “…inborn, all around intellectual ability … inherited, but not due to teaching or training… uninfluenced by industry or zeal^37^, cited by Nisbett^33^.” Similarly, Jensen maintains that “… the means for changing intelligence per se lie in the province of biology rather than psychology or education^38^.” Herrnstein and Murray^36^ expresses a parallel assessment that the differences in intelligence are mostly at the hand of nature and there is little that government policies could change. This position tends to dismiss the influence of environmental factors and views cognitive ability largely as a genetic trait.

In the meanwhile, mainstream behavior geneticists, relying on twins and other blood relatives, show a heritability estimate of cognitive ability that ranges between 0.2 to 0.8^9,10^ and a shared non-genetic factor that quickly reduces to zero after maturity^9^. These findings have set a benchmark for ensuing work to be compared and assessed. Nevertheless, twin studies’ estimates at population level and the usual lack of extensive SES measures make them inadequate for understanding how heredity and SES jointly and interactively mold human cognitive ability.

Advances in molecular genetics over the past two decades have brought forth DNA data at individual level. The efforts linking DNA variation to cognitive ability began in earnest in the early 2000s. By then, it is evident that cognitive ability is a complex trait subject to the influence of a large number of genes each with a tiny effect^39^. The challenge to find specific genes for cognitive ability is enormous. A human genome consists of millions of genetic variants or sections of DNA that may differ across individuals. Testing whether each one of them predicts ability and setting the critical value for significance at the conventional level of 0.05 would by chance generate a large number of false positives. Although the genome is large, it is finite. The solution is to set a stringent critical value of 5 × 10^−8^ for significance and to request a replication of a discovered genetic variant in an independent data source. Initial successes of the GWAS employing thousands of individuals are performed on human traits such as type 2 diabetes and body mass index (BMI)^40,41^. The number of GWAS-identified genetic loci are small but tended to be replicated.

It soon became clear that by far the single most important factor in GWAS is sample size. The GWAS of educational attainment in 2018 assembles 1.1 million individuals and reports 1,271 independent SNPs associated with years of education at the genome-wide significance level of 5 × 10^−8^ ^13^. The polygenic score (PGS) for educational attainment constructed from all common SNPs in the GWAS reports an *R*^*2*^ of about 12% using data from Add Health. Many of the genetic loci are implicated in biological pathways that play a role during prenatal brain development.

The 2018 GWAS of cognitive ability^14^ employs 269,867 individuals and identifies 205 independent genome-wide significant loci. The analysis data for the GWAS are assembled from more than a dozen cohorts, and different cohorts tend to use different cognitive tests. In spite of the differences in form, cognitive tests are all based on the common underlying fluid intelligence or the Spearman’s g, which is likely to have a large impact on multiple domains of cognitive functioning. The g factors extracted from different cognitive tests are highly correlated^42,43^, substantiating the approach used in the GWAS.

The GWAS-identified genes are mostly expressed in brain tissues. The genetic correlation between intelligence and education is estimated to be about 0.70 with *p* = 2.5 × 10^−287 ^^44^. The genetic correlation computes the correlation between the genetic influence behind cognitive ability and that behind education, suggesting that the education-based GWAS-identified genetic variants ought to be reasonable predictors of cognitive ability. In the present analysis, the latest findings of GWAS for cognitive ability and educational attainment are used to construct the genomic measures to be included in models predicting verbal ability. See additional discussion in Supplementary Materials: I. Genetic terms and concepts.

Gene-environment (G×E) interaction analysis in this context examines how genotype exacerbates or suppresses the effect of SES or vice versa. Our G×E-interaction hypothesis predicts that a favorable SES context enhances the positive effect of genomic measures of verbal ability. The reasoning goes that a favorable SES context would help realize the genetic potential of an individual’s ability. Previous G×E-interaction work on cognitive development using twin data generated mixed results. Several studies report evidence supporting our hypothesis^45–47^; however, an exceptionally large study failed to find any significant G×E-interaction effect^48^. G×E-interaction analysis is methodologically challenging. To mitigate the threat of multiple testing, our analysis uses a principal component as a summary measure for the multiple dimensions of SES. We have also considered a number of issues raised in recent literature on G×E-interaction studies including the lack of sufficient controls^49^, coarsened outcome variables and sample selection^50^, and the collider bias^51^.

**Fig. 1:**
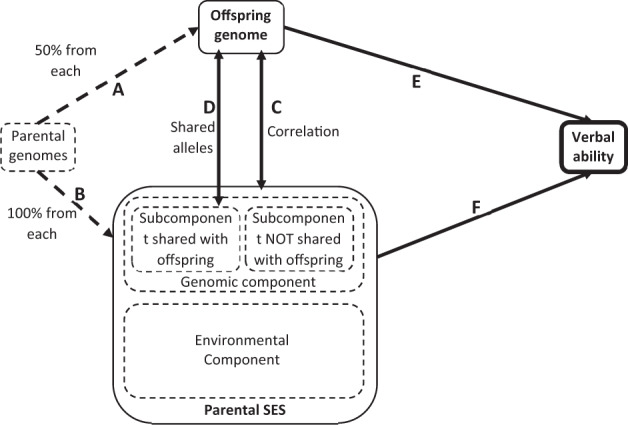
Conceptual model of how parental genome, parental SES and offspring genome influence verbal ability. *(An arrowed line indicates a causal effect; a double-arrowed line indicates a correlation. Solid lines represent observed relationships while interrupted lines represent unobserved relationships*). **a** While offspring genome receives 50% of genetic alleles from each parent’s genome randomly (path A), parental SES is subject to the influence of all parental alleles (100% from each parent, (path B). **b** Offspring genome and parental SES are correlated (path C) due to the shared alleles between offspring genome and the subcomponent of the genomic component in parental SES (path D). **c** The same set of alleles from offspring and parents act on the subcomponent shared between parents and offspring; the subcomponent not shared with offspring is only acted upon by parental alleles not transmitted to offspring. The two subcomponents are hypothesized to be about the same. **d** In regression of verbal ability, the effects of offspring genome and parental SES are expected to be reduced because of the correlation. **e** The size of reduction in the offspring-genome effect may reflect the size of an overestimate in a regression in which parental SES is not included. **f** The effect of parental SES is reduced because the inclusion of offspring genome removes about one half of the entire genomic component in parental SES. The total size of the genetic component in parental SES is reasoned to be about twice as large as the reduction in the effect of parental SES when offspring genome is included.

Family SES has a genomic component. Kong et al.^16^ measures the genomic component in family SES directly by transmitted parental alleles (T) and non-transmitted parental alleles (NT). “T” refers to transmitted from parents to children. Kong et al. estimates the effects of T and NT on children’s educational attainment. Both effects are highly significant. The estimated NT effect confirms the genomic component in parental SES (Fig. 1). The estimated T effect is about three times as large as that of NT; but this is an upward-biased effect of children’s genome. Kong et al. concludes that the unbiased effect of children’s genome is T-NT.

Without parental genomic data, our analysis is unable to estimate the effect of NT directly. Instead, our analysis capitalizes on offspring genome and parental SES. Figure 1 describes our conceptual model that illustrates what may be learned from a regression of verbal ability on parental SES and offspring genome. In the figure, an arrowed line indicates a causal effect, a double-arrowed line indicates a correlation; solid lines represent observed relationships while interrupted lines represent unobserved relationships. Parental SES is traditionally viewed as environmental. Current literature points to a substantial genomic component in parental SES^16,52,53^. Parental genomes likely shape family SES such as education, occupation, income, and wealth at the family level as well as the neighborhood the family lives in and the schools the children attend, which is the genomic component in parental SES.

Parental SES could be decomposed into an environmental component and a genomic component. The latter originates from parental genomes. Fifty percent of each parent’s alleles transmit to offspring; but 100% of each parent’s alleles act on parental SES. This observation suggests that (1) a common origin of family SES and offspring genome induces a correlation between the two (path C), (2) the essence of path C is Correlation path D generated by the same set of alleles shared by offspring and parents, (3) the subcomponent not shared with offspring in the genomic component is acted upon by parental alleles that are not transmitted to offspring, (4) the two subcomponents are hypothesized to be about the same since each subcomponent is acted upon by about 50% of total parental alleles, implying that the size of the genomic component is twice as large as the subcomponent shared with offspring; and (5) we most likely have not exhausted the genomic confounding of SES influences.

The recognition of these subtleties when parental SES and offspring genome data are available leads to three payoffs even in the absence of parental genomic data. First, we have the opportunity to correct for the upward bias in the effect of offspring genome. The usually-reported genomic effect includes the effect of offspring genome as well as the impact of the genomic subcomponent in parental SES shared between parents and offspring. This subcomponent could be considered the upward bias in the typically-estimated offspring genome effect. The upward bias could be corrected by including parental SES in the model. Second, including offspring genome and parental SES in the same model provides an estimated size of the genomic component in parental SES, which we hypothesize to be about twice as large as the reduction in the parental SES effect when estimated simultaneously with offspring genome. Third, our analysis yields an estimated size of the environmental component of parental SES, which may be calculated as enviSES = Parental SES-2(ReducedSES), where enviSES is the environmental SES, Parental SES is the estimate from a model including only parental SES, and ReducedSES is the reduced size of parental SES when offspring genome is added.

Ethnic samples complicate genetic analysis. Our analysis is based on European Americans (whites) and Hispanic European Americans (Hispanic whites). We provide results separately for whites, Hispanic whites and the combined sample of the two. African Americans, Asian Americans, Native Americans and Hispanic African Americans are excluded because the published GWAS studies are mostly based on individuals of European descent including the GWAS that identified the genetic loci associated with educational attainment and cognitive ability^54^. Substantial genomic differences in individuals from different continents have been known since 1990s^55^. The partial overlap between the European genetic variants, and those of Africans and Asians implies that the related GWAS must have missed a substantial number of genetic loci among the ethnic minorities that are associated with educational attainment and cognitive ability. This, in turn, suggests that the GWAS findings do not apply to racial minorities as well as they do to European Americans^56^.

Do problem behavior and general health, especially mental health affect cognitive measures? Over the past decades, there has been a sustained effort to examine how educational attainment and educational achievement are impacted by problem behavior^57–61^ and health^62^, especially mental health and stress^60,61,63–66^. In this project, we test the impact of adding delinquency, self-reported health and depression to the model that already has SES and offspring genome as predictors.

This research has one overall objective and five hypotheses. The present research has an overarching objective of estimating the relative contribution of offspring genome and parental SES to verbal ability and five specific hypotheses. The first two establish in our analysis data what is known previously. The first hypothesizes that collectively, SES significantly predicts verbal ability (path F in Fig. 1) when offspring genome is not included in the model. The second hypothesizes that the two PGSs for education and ability significantly predict verbal ability (path E) without parental SES in the model. The third hypothesizes that the inclusion of SES reduces the effect size of the two PGSs. The fourth hypothesizes that the inclusion of the PGSs reduces the effect size of SES. The fifth is a G×E-interaction hypothesis testing whether favorable SES increases the positive effect of the PGSs.

## Results

### Main-effect models

Table 1 shows the means and the standard deviations of the continuous variables, and the percentage distribution of categorical variables used in the analysis for whites, Hispanic whites and the combined whites and Hispanic whites. The heritability is estimated to be 0.38; this estimate is based on a small sample of 73 pairs of MZ twins and 92 pairs of DZ twins in our analytic Add-Health sample.

**Table 1:** Descriptive statistics for the Add-Health data.

Variables	Whites (*N* = 5820)	Hispanic Whites (*N* = 1374)	Whites and Hispanic Whites (*N* = 7194)
	Mean or % (S.D.)	Mean or % (S.D.)	Mean or % (S.D.)
PVT Standardized Score (Verbal Ability)	104.5 (11.8)	94.7 (17.3)	102.7 (13.6)
Ability Polygenic Scores (PGS)			
PGS for education	0.0 (1.0)	0.0 (1.0)	0.0 (1.0)
PGS for cognitive ability	0.0 (1.0)	0.0 (1.0)	0.0 (1.0)
SES context			
Mother’s education			
Less than high school	11.4	36.4	16.1
High school/some college	59.1	43.2	54.1
≥College graduation	26.8	12.4	25.1
Missing	2.8	8.0	3.8
Father’s education			
Less than high school	11.0	37.7	16.0
High school/some college	56.5	39.8	53.4
≥College graduation	26.3	12.2	23.6
Missing	6.2	10.3	7.0
Mother’s occupation			
None and other	24.3	33.8	26.1
Manual or blue collar	15.1	25.3	17.0
Sales/service/administrative	26.2	19.3	24.9
Professional or managerial	29.2	16.6	26.8
Missing	5.2	5.1	5.2
Father’s occupation			
None and other	11.8	14.6	12.3
Manual or blue collar	36.2	42.5	37.4
Sales/service/administrative	6.3	3.1	5.7
Professional or managerial	25.7	13.3	23.4
Missing	20.1	26.4	21.3
Household income			
0–20 percentile	9.5	20.5	11.6
20–40 percentile	12.4	17.6	13.4
40–60 percentile	18.5	15.6	17.9
60–80 percentile	21.2	12.2	19.5
80–100 percentile	20.9	7.5	18.4
Missing	17.6	26.6	19.3
Family structure			
2 Biological parents	53	47	51
Sibling size			
No sibling	4.1	3.6	4.0
1 to 2 sibling(s)	55.2	41.4	52.6
3 to 5 siblings	27.0	33.7	28.2
6 to 20 siblings	13.6	21.2	15.1
Other and missing	0.1	0.2	0.1
Neighborhood disadvantages	0.0 (1.0)	0.0 (0.9)	0.0 (1.0)
In School	58	54	57
Demographics			
Age	18.9 (3.6)	19.3 (3.6)	18.9 (3.6)
Gender			
Female	53	49	52
Male	47	51	48
Race and Ethnicity			
White	–	–	81.1
Hispanic	–	–	18.9
Immigration status			
US Born	71	53	67
Speaking English at Home	100	55	91
Heritability: MZ (73 pairs), DZ (92 pairs)			0.38

We organize our findings by Table 2 (SES results), Table 3 (genomic results), and Table 4 (SES and genomic results) so that we could observe the impact of SES and genome. Table 2 presents the coefficients and their standard errors of SES context and other covariates from multilevel models of verbal ability for whites, Hispanic whites and the two combined. The model is a 3-level model of verbal ability (Eq. 1). The two random effects at the individual and family levels are highly statistically significant. The OLS *R*^*2*^ is estimated to range from 12.7% to 19.9% across the different samples.

**Table 2:** Multilevel models of Wave-1 and 3 verbal ability showing coefficients (standard errors) of SES context.

Predictors	Whites	Hispanic Whites	Whites and Hispanic Whites
	SES	SES	SES
	β (S.E.)	β (S.E.)	β (S.E.)
SES context			
Mother’s education			
Less than high school	–	–	–
High school/some college	3.29 (0.56)***	0.49 (1.37)	2.63 (0.52)***
≥College graduation	6.10 (0.69)***	4.73 (1.99)*	5.65 (0.67)***
Missing	−0.53 (1.05)	−1.90 (2.32)	−1.07 (0.96)
Father’s education			
Less than high school	–	–	–
High school/some college	1.87 (0.54)***	3.39 (1.34)*	2.09 (0.51)***
≥College graduation	2.16 (0.66)**	0.80 (1.90)	1.96 (0.64)**
Missing	0.94 (0.94)	0.51 (2.28)	0.59 (0.89)
Mother’s occupation			
Manual or blue collar	–	–	–
None and others	−0.04 (0.39)	0.67 (0.97)	0.03 (0.37)
Sales/service/administrative	0.49 (0.39)	0.01 (1.19)	0.44 (0.39)
Professional or managerial	0.35 (0.41)	0.77 (1.28)	0.42 (0.41)
Missing	−0.37 (0.82)	0.07 (2.05)	−0.16 (0.78)
Father’s occupation			
Manual or blue collar	–	–	–
None and others	−0.06 (0.40)	−0.31 (1.08)	−0.14 (0.39)
Sales/service/administrative	1.27 (0.52)*	2.73 (2.02)	1.51 (0.54)**
Professional or managerial	2.40 (0.34)***	2.57 (1.21)*	2.50 (0.34)***
Missing	0.62 (0.40)	0.54 (1.07)	0.45 (0.39)
Household Income W1			
0–20 percentile	–	–	–
20–40 percentile	1.16 (0.59)*	3.91 (1.33)**	1.91 (0.54)***
40–0 percentile	2.24 (0.56)***	5.83 (1.40)***	2.91 (0.53)***
60–80 percentile	2.90 (0.56)***	4.70 (1.62)**	3.31 (0.54)***
80–100 percentile	3.46 (0.59)***	6.69 (1.91)***	3.88 (0.57)***
Missing	1.15 (0.56)*	−0.30 (1.20)	0.84^+^ (0.51)
2 biological parents W1	0.09 (0.34)	−1.17 (0.95)	−0.17 (0.33)
Sibship size			
No sibling	–	–	–
1 to 2 siblings	−2.22 (0.68)**	−1.81 (2.14)	−2.14 (0.68)**
3 to 5 siblings	−2.57 (0.71)***	−4.12 (2.16)^+^	−2.84 (0.70)***
6 to 20 siblings	−3.04 (0.76)***	−5.37 (2.24)*	−3.62 (0.74)***
Other and Missing	1.14 (4.20)	0.03 (10.03)	0.71 (3.95)
Neighborhood Disadvantages	−0.41 (0.10)***	−1.0 (0.32)5***	−0.60 (0.10)***
In School	1.54 (0.31)***	0.97 (1.03)	1.47 (0.32)***
Demographics			
Age	0.34 (0.04)***	0.63 (0.15)***	0.40 (0.04)***
Female	−1.20 (0.26)***	−0.70 (0.76)	−1.10 (0.26)***
Immigration status			
US Born	1.65 (0.29)***	3.58 (0.79)***	2.00 (0.28)***
Speaking English Home	6.35 (2.05)**	3.63 (0.91)***	4.60* (0.61)**
Race and Ethnicity			
White	–	–	–
Hispanic	–	–	−4.41 (0.43)***
Constant	84.34 (2.42)***	76.47 (4.12)***	84.98 (1.43)***
Random effects			
$\sigma _u^2$, family-level	1.78 (0.05)***	2.40 (0.04)***	1.94 (0.04)***
$\sigma _v^2$, person-level	1.74 (0.05)***	−7.09 (0.65)***	1.69 (0.06)***
$\sigma _e^2$, wave-level	2.00 (0.01)***	2.46 (0.02)***	2.13 (0.01)***
Model-level parameters			
(−2) Log-Likelihood	77,794	20,004	98,618
Person-observations	10,401	2421	12,822
Number of Families	5208	1276	6466
Number of Persons	5820	1374	7194
OLS *R*^*2*^	0.127	0.140	0.199

***p < 0.001; **p < 0.01; *p < 0.05; ^+^*p* < 0.1.

**Table 3:** Multilevel models of Wave-1 and 3 verbal ability showing coefficients (standard errors) of ability-related PGSs.

Predictors	Whites	Hispanic Whites	Whites and Hispanic Whites
	PGS	PGS	PGS
	β (S.E.)	β (S.E.)	β (S.E.)
Ability PGSs			
PGS for education	2.32 (0.14)***	1.91 (0.44)***	2.27 (0.15)***
PGS for IQ	0.31 (0.14)*	0.89 (0.43)*	0.44 (0.14)**
Population admixture	Omitted^#^	Omitted^#^	Omitted^#^
Constant	102.8 (0.74)***	100.6 (1.06)***	100.7 (0.43)***
Random effects	Omitted^#^	Omitted^#^	Omitted^#^
Model-level parameters	Some omitted^#^	Some omitted^#^	Some omitted^#^
Number of persons	5820	1374	7194
(−2)Log-Likelihood	78,396	20,170	99,396
OLS *R*^*2*^	0.042	0.080	0.126

^#^“Omitted” indicates that the parameters are very similar to those in previous models and omitted to avoid redundancy.

***p < 0.001; **p < 0.01; *p < 0.05; ^+^*p* < 0.1.

**Table 4:** Multilevel models of Wave-1 and 3 verbal ability showing coefficients (standard errors) of SES context and ability-related PGSs.

Predictors	Whites	Hispanic Whites	Whites and Hispanic Whites
	SES + PGS	SES + PGS	SES + PGS
	β (S.E.)	β (S.E.)	β (S.E.)
Ability PGSs			
PGS for education	1.72 (0.14)***	1.62 (0.42)***	1.73 (0.14)***
PGS for IQ	0.25 (0.13)^+^	1.00 (0.41)*	0.41 (0.13)**
SES context			
Mother’s Education			
Less than high school	–	–	–
High school/some college	2.96 (0.55)***	−0.22 (1.36)	2.21 (0.52)***
≥College graduation	5.56 (0.68)***	3.45 (1.96)^+^	4.98 (0.66)***
Missing	−0.61 (1.04)	−2.22 (2.28)	−1.18 (0.94)
Father’s Education			
Less than high school	–	–	–
High school/some college	1.87 (0.53)***	3.25 (1.32)*	2.00 (0.50)***
≥College graduation	2.04 (0.65)**	0.81 (1.88)	1.81 (0.63)**
Missing	0.77 (0.93)	0.45 (2.25)	0.43 (0.88)
Mother’s occupation			
Manual or blue collar	–	–	–
None and others	−0.12 (0.39)	0.40 (0.95)	−0.06 (0.37)
Sales/service/administrative	0.43 (0.39)	−0.34 (1.18)	0.27 (0.38)
Professional or managerial	0.31 (0.41)	0.60 (1.27)	0.32 (0.40)
Missing	−0.37 (0.81)	−0.07 (2.02)	−0.25 (0.77)
Father’s occupation			
Manual or blue collar	–	–	–
None and others	0.00 (0.40)	−0.57 (1.07)	−0.17 (0.38)
Sales/service/administrative	1.19 (0.52)*	1.86 (2.00)	1.33 (0.53)*
Professional or managerial	2.22 (0.33)***	1.97 (1.19)^+^	2.27 (0.34)***
Missing	0.54 (0.40)	0.09 (1.05)	0.30 (0.38)
Household Income Wave 1			
0–20 percentile	–	–	–
20–40 percentile	0.86 (0.58)	3.94 (1.30)**	1.79 (0.53)***
40–60 percentile	1.87 (0.55)***	5.73 (1.38)***	2.70 (0.52)***
60–80 percentile	2.45 (0.56)***	4.35 (1.58)**	3.02 (0.53)***
80–100 percentile	2.84 (0.58)***	6.13 (1.87)**	3.45 (0.56)***
Missing	0.79 (0.55)	0.10 (1.18)	0.77 (0.50)
2 Biological Parents Wave 1	0.03 (0.33)	−1.09 (0.93)	−0.20 (0.32)
Sibship size			
No sibling	–	–	–
1 to 2 siblings	−2.24 (0.67)***	−1.21 (2.10)	−2.06 (0.67)**
3 to 5 siblings	−2.64 (0.70)***	−3.03 (2.13)	−2.70 (0.69)***
6 to 20 siblings	−2.99 (0.75)***	−3.81 (2.21)^+^	−3.25 (0.73)***
Other and missing	0.86 (4.14)	−2.44 (9.81)	−0.29 (3.88)
Neighborhood disadvantages	−0.40 (0.10)***	−1.01 (0.32)**	−0.59 (0.10)***
In School	1.42 (0.31)***	0.74 (1.02)	1.34 (0.31)***
Demographics			
Age	0.32 (0.04)***	0.59 (0.14)***	0.38 (0.04)***
Female	−1.16 (0.26)***	−0.92 (0.75)	−1.07 (0.25)***
Immigration status			
US born	1.61 (0.28)***	3.30 (0.78)***	1.90 (0.28)***
Speaking English Home	6.80 (2.06)***	3.17 (0.93)***	4.13 (0.61)***
Race and Ethnicity			
White	–	–	–
Hispanic	–	–	−1.04 (0.64)
Population admixture	Omitted^#^	Omitted^#^	Omitted^#^
Constant	82.6 (2.48)***	80.1 (4.23)***	84.1 (1.46)***
Random effects	Omitted^#^	Omitted^#^	Omitted^#^
Model-level parameters	Some omitted^#^	Some omitted^#^	Some omitted^#^
Number of persons	5820	1374	7194
(−2)Log-Likelihood	77,606	19,946	98,372
OLS *R*^*2*^	0.147	0.166	0.219
* R*^*2*^ without PGSs, with PCAs	0.128	0.154	0.204
* R*^*2*^ from net PGS effects	0.019	0.012	0.015

^#^“Omitted” indicates that the parameters are very similar to those in previous models and omitted to avoid redundancy.

***p < 0.001; **p < 0.01; *p < 0.05,^+^*p* < 0.1.

The models presented in Table 2 are traditional social-science models that include a full set of SES predictors without any genomic measure. SES context proves important. All social-contextual characteristics except for mother’s occupation significantly and simultaneously predict verbal ability in direction consistent with expectation. For example, in the combined sample, individuals whose mothers have at least a college degree score 5.65 points higher on verbal ability than those whose mothers have an education less than high school. Those whose fathers have at least a college degree score 1.96 points higher than those whose fathers have an education less than high school. Those whose fathers hold a professional and managerial job are about 2.50 points higher than those whose fathers hold a manual and blue-collar job. Individuals living in a household in the top 20% income group score 3.88 points higher than those in the lowest 20% income group. Individuals living in a household with 3 to 5 siblings score 2.84 points lower than those who are the only child in a family. An increase in one standard deviation in the index of the neighborhood disadvantage is associated with a decrease of 0.60 point of the verbal ability score. Those who are in a school session when taking the test score about 1.47 points higher than those who are not in a school session.

Table 3 presents the coefficients and their standard errors of ability PGSs from multilevel models of verbal ability. Among whites and Hispanic whites, one standard deviation of the education PGS is associated with 2.27 points of verbal ability, and one standard deviation of the ability PGS is associated with 0.44 points of verbal ability. These results establish the importance of the two PGSs.

The models in Table 4 include all SES measures as well as the two ability PGSs. These models could be compared with the models in Table 2 to assess the impact of the two PGSs on the effects of parental SES. Essentially, all SES measures that are statistically significant in the model without the two PGSs have remained significant in the model with the PGSs. As expected, the inclusion of the PGSs attenuates the SES effect sizes. In most cases, the inclusion of the PGSs reduces the SES coefficients in models in Table 2 by about 10–15%. For example, in Table 2, the two categories of mother’s education “high-school graduation/some college” and “at least college degree”, respectively, are associated with 2.63 and 5.65 additional points in the verbal ability score relative to “less than high school” in the model without the PGSs. The two comparable estimates in the model with the two PGSs in Table 4 are 2.21 and 4.98, which represent reductions of 15.9% and 11.8%, respectively. The models in Table 4 could also be compared with the models in Table 3 to assess the impact of parental SES on the two PGS estimates. The effects of both PGSs are reduced, with reductions of 23.6% and 6.8%, respectively, for the education PGS and the ability PGS when parental SES is included in the model.

The incremental or “net” *R*^*2*^s in Table 4 are the *R*^*2*^s due to the two PGSs above and beyond SES. These *R*^*2*^s may be considered a measure of the impact of offspring genome on verbal ability. Such an incremental *R*^*2*^ could be obtained by subtracting the *R*^*2*^ of a model containing SES and the principal components (PCAs) from the *R*^*2*^ in the corresponding model in Table 4. The incremental *R*^*2*^ or “net” *R*^*2*^ in the combined sample is 1.5%. A comparison of another direction can be made between the *R*^*2*^ (21.9%) in Table 4 and that (12.6%) of Table 3. The incremental *R*^*2*^ of 21.9–12.6 = 9.3% due to parental SES is apparently much larger than the incremental *R*^*2*^ of 21.9–20.4 = 1.5% due to the two PGSs, but this *R*^*2*^ of 9.3% needs to be interpreted carefully.

Supplementary table 1 presents findings from three models based on 719 full siblings and DZ twins in Add Health. The analysis tests the robustness of the effects of ability PGSs when controlling for the genomic impact in parental SES^17^. The first of the three models estimates the effects of the two PGSs alone; the second adds observed parental SES; the third estimates a sibling-fixed-effect model that controls for all shared effects among the siblings. Consistent with the findings from our full-sample analysis (Table 4), adding observed parental SES reduces the effects of the two PGSs. The fixed-effects model reduces further the effects of the PGSs. The PGS effects in the fixed-effects model remain significant and substantial in size.

The models in Table 5 add the Wave-1 verbal ability as a predictor to the model that uses SES measures and the two PGSs to predict the Wave-3 verbal ability (Eq. 2). The Wave-1 verbal ability was measured about seven years before the Wave-3 verbal ability was measured. A total of 2,176 individuals are excluded in the analysis because an individual must contribute two measures of verbal ability to be included in this analysis. Little surprise that the PVT score at Wave 1 is highly predictive of the PVT score at Wave 3. In the combined sample, an increase of one point in the Wave-1 PVT score is associated with an increase of about 0.49 points in the Wave-3 PVT score. The effect of one standard deviation of the education PGS is reduced from 1.73 points in the combined sample in Table 4 to 0.51 points in Table 5. The ability PGS has lost its statistical significance.

**Table 5:** Multilevel models of Wave-3 verbal ability showing coefficients (standard errors) of SES context and ability-related PGSs conditional on Wave-1 verbal ability.

Predictors	Whites	Hispanic Whites	Whites and Hispanic Whites
	SES + PGS	SES + PGS	SES + PGS
	β (S.E.)	β (S.E.)	β (S.E.)
Verbal Ability at Wave 1	0.49 (0.01)***	0.48 (0.03)***	0.49 (0.01)***
Ability PGSs			
PGS for education	0.38 (0.14)**	1.00 (0.50)*	0.51 (0.15)***
PGS for IQ	−0.02 (0.13)	0.78 (0.50)	0.13 (0.14)
SES context			
Years of Education by Wave 3	0.74 (0.08)***	0.90 (0.26)***	0.78 (0.08)***
Mother’s Education			
Less than High School	–	–	–
High school/some college	1.08 (0.61)^+^	0.87 (1.83)	1.14 (0.61)^+^
≥College graduation	1.64 (0.77)*	2.81 (2.67)	1.77 (0.79)*
Missing	0.42 (1.13)	−4.62 (3.05)	−0.79 (1.10)
Father’s Education			
Less than High School	–	–	–
High school/some college	0.09 (0.61)	0.95 (1.82)	0.33 (0.61)
≥College graduation	0.02 (0.76)	0.59 (2.60)	0.27 (0.78)
Missing	0.08 (1.08)	5.53 (3.11)^+^	1.17 (1.08)
Mother’s Occupation Wave 1			
Manual or blue collar	–	–	–
None and others	−0.22 (0.43)	0.98 (1.22)	−0.55 (0.93)
Sales/service/administrative	−0.11 (0.45)	1.68 (1.54)	0.11 (0.43)
Professional or managerial	−0.30 (0.47)	2.46 (1.68)	0.29 (0.46)
Missing	−0.05 (0.93)	−2.92 (2.73)	0.17 (0.49)
Father’s Occupation Wave 1			
Manual or blue collar	–	–	–
None and others	0.21 (0.41)	0.59 (1.32)	−0.27 (0.45)
Sales/service/administrative	−1.07 (0.58)^+^	3.18 (2.80)	0.35 (0.42)
Professional or managerial	0.31 (0.37)	0.08 (1.57)	−0.69 (0.63)
Missing	−0.35 (0.43)	0.52 (1.37)	0.30 (0.40)
Household Income at Wave 1			
0–20 percentile	–	–	–
20–40 percentile	−0.24 (0.57)	1.34 (1.56)	0.20 (0.56)
40–60 percentile	−0.53 (0.54)	2.04 (1.68)	0.02 (0.55)
60–80 percentile	0.09 (0.55)	−2.15 (1.91)	−0.03 (0.57)
80–100 percentile	0.10 (0.59)	3.42 (2.28)	0.46 (0.60)
Missing	−0.65 (0.55)	−0.53 (1.46)	−0.64 (0.54)
2 Biological Parents at Wave 1	−0.61 (0.34)+	−0.94 (1.15)	−0.81 (0.35)*
Sibling size			
No sibling	–	–	–
1 to 2 siblings	0.55 (0.65)	−0.78 (2.41)	0.23 (0.69)
3 to 5 siblings	0.32 (0.68)	−1.42 (2.44)	−0.08 (0.71)
6 to 20 siblings	−0.08 (0.73)	−3.60 (2.56)	−1.02 (0.76)
Other and Missing	3.79 (4.38)	−4.84 (10.66)	2.40 (4.17)
Neighborhood disadvantages W1	−0.60 (0.15)***	0.09 (0.53)	−0.44 (0.16)**
Neighborhood disadvantages W3	−0.02 (0.13)	−0.49 (0.52)	−0.08 (0.14)
Demographics			
Age (Wave 3)	0.43 (0.08)***	−0.25 (0.27)	0.30 (0.08)***
Female	−0.44 (0.26)^+^	0.09 (0.92)	−0.34 (0.27)
Immigration status			
US born	0.70 (0.29)*	−0.27 (0.98)	0.43 (0.30)
Speaking English Home	0.51 (2.05)	−1.03 (1.14)	−0.55 (0.66)
Race and Ethnicity			
White	–	–	–
Hispanic	–	–	−0.87 (0.68)
Population admixture	Omitted^#^	Omitted^#^	Omitted^#^
Constant	32.4 (3.03)***	46.0 (7.57)***	35.9 (2.39)***
Random effects	Omitted^#^	Omitted^#^	Omitted^#^
Model-level parameters	Some omitted^#^	Some omitted^#^	Some omitted^#^
−2 Log-Likelihood	32,737	8550	41,922
Number of Persons	4579	1047	5626
OLS *R*^*2*^	0.405	0.296	0.407

^#^“Omitted” indicates that the parameters are very similar to those in previous models and omitted to avoid redundancy.

***p < 0.001; **p < 0.01; *p < 0.05; ^+^*p* < 0.1.

Conditional on the Wave-1 PVT, the effects of SES are expected to be largely washed out. This is what we observe. Most of the SES measures no longer significantly predict verbal ability at Wave 3. The two remarkable exceptions are years of education by Wave 3 and neighborhood disadvantage at Wave 1. The former is significantly predictive of Wave-3 PVT with a *P* value of 0.001, with each additional year of schooling associated with about 0.78 point of verbal ability score. It is neighborhood disadvantage in earlier life at Wave 1 rather than at Wave 3 that has turned out to be negatively associated with verbal ability.

Supplementary Tables 2–5 provide a set of findings parallel to those in Tables 2–5. The difference is that in Supplementary Tables 2–5, the SES measures are represented by a single summary score obtained from the first principal component of a principal component analysis. In the models, the summary score is always highly significant. The results convey a comparable story as that told by using specific SES measures.

### G×E interaction and other models

Table 6 shows the main effects and G×E-interaction effects on verbal ability between the PGSs and a PCA summary score of SES. A likelihood ratio test shows that the G×E-interaction model (−2LogL = 67,058; df = 24) has significantly more explanatory power than the main effects model (−2LogL = 67,064; df = 22) at the level of 5% with two degrees of freedom. The interdependence between the education PGS and SES is positive, indicating that a favorable SES environment would enhance the effect of offspring genome.

**Table 6:** Multilevel models of Wave-1 and 3 verbal ability showing G×E-interaction coefficients (standard errors) between an SES principal component and the two ability-related PGSs.

Predictors	Main-effect Model	G×E-Interaction Model
	Whites and Hispanic Whites	Whites and Hispanic Whites
	SES	SES
	β (S.E.)	β (S.E.)
Ability PGSs		
PGS for education	1.88 (0.16)***	1.88 (0.16)***
PGS for IQ	0.32 (0.16)*	0.32 (0.16)*
SES PC	3.08 (0.16)***	3.08 (0.16)***
PGS × SES		
PGS for Education*SES PC	–	0.36 (0.15)*
PGS for IQ*SES PC	–	−0.21 (0.15)
Demographics		
Age	0.21 (0.03)***	0.21 (0.03)***
Female	−1.18 (0.30)***	−1.19 (0.30)***
Immigration status		
US Born	1.80 (0.33)***	1.83 (0.33)***
Speaking English Home	5.04 (0.78)***	5.08 (0.78)***
Race and Ethnicity		
White	–	
Hispanic	−1.43 (0.78)^+^	−1.41 (0.78)^+^
Population admixture	Omitted^#^	Omitted^#^
Constant	94.4 (1.07)***	94.2 (1.08)***
Random effects	Omitted^#^	Omitted^#^
Model-level parameters	Some omitted^#^	Some omitted^#^
−2LogLikelihood	67,064	67,058
Number of Persons	4566	4566
OLS *R*^*2*^	0.19	0.19

^#^“Omitted” indicates that the parameters are very similar to those in previous models and omitted to avoid redundancy.

***p < 0.001; **p < 0.01; *p < 0.05; ^+^*p* < 0.1.

Supplementary Table 7 tests additional effects of general health, mental health and delinquency behavior on verbal ability when parental SES and the two PGSs are in the model. Out of the three, only mental health significantly predicts verbal ability with an expected negative impact.

We carried out the MICE analysis, an alternative way of addressing missing values and the analysis yielded a very similar set of results as those in the analysis that codes missing values as a separate category.

## Discussion

To summarize, our analysis regresses verbal ability on two ability PGSs and parental SES in an Add-Health sample of 7194 individuals. Our first two hypotheses concerning the effects of parental SES alone (Table 2) and the effects of the two PGSs alone (Table 3) are supported. The traditional sociological model of SES context predicts verbal ability well (Table 2). Of the two PGSs, the education PGS has a much more robust prediction of verbal ability than the intelligence PGS; the much larger discovery sample size for the former could be one of the explanations. After demographic variables and immigration status are adjusted, mother’s education, father’s education, father’s occupation, household income, sibship size, neighborhood disadvantage, and in school over the past year are all significantly and simultaneously associated with verbal ability. Only mother’s occupation is an exception. The two PGSs significantly and simultaneously predict verbal ability (Table 3). These SES and PGS estimates are “nominal” effects in the sense that both the PGS and SES estimates are upwardly biased because of the impact of parental genomes (Fig. 1).

Key evidence supporting Hypotheses 3 and 4 is revealed after comparing Table 4 with Tables 2, 3. When SES and PGSs are included in the same model, both continue to significantly predict verbal ability; but the estimates are reduced. The reduction in the education PGS is more than 20% and the reduction in the ability PGS is about 7%. These reductions may be interpreted as the upward biases when parental genomic effects are not controlled. The reduction in the SES effects is about 10–15%. These percentages may be interpreted as one half of the effect in parental SES induced by parental genomes. In other words, about 20–30% of parental SES effects is genomic from parental genomes. Sample variation in a particular study is likely a major source of the variation in these estimates. But the empirical evidence does seem to support an important role of parental genomes in parental SES.

The relative contribution of SES and genomes to verbal ability may be assessed by incremental *R*^*2*^s. GWAS studies routinely report *R*^*2*^ as an indicator of the amount of explanatory power they have secured in explaining an outcome^13^. Supplementary Table 6 lists the *R*^*2*^s of models with different combinations of parental SES, two ability PGSs and the PCAs. The model in Table 4 yields an *R*^*2*^ of 21.9% reflecting the effects of offspring genome, parental SES and population stratification. Incremental *R*^*2*^s could be derived from comparing the *R*^*2*^s across the models. The incremental *R*^*2*^ of offspring genome measures the explanatory power of offspring genome on verbal ability above and beyond SES. Similarly, the incremental *R*^*2*^ of SES measures the overall explanatory power of SES in addition to offspring genome. To estimate the incremental *R*^*2*^ from the influence of offspring genome, we deduct from 21.9% the *R*^*2*^ of 20.04% in the comparison model that includes parental SES and PCAs; this yields an *R*^*2*^ of 1.5%. This incremental *R*^*2*^ is from offspring genome alone and the impact of the genomic component in parental SES is excluded. Importantly, this *R*^*2*^ is also net of the explanatory power due to population stratification. To estimate the contribution of parental SES, we deduct from 21.9% the *R*^*2*^ of 12.6% in model 3 that includes the two PGSs and the PCAs, obtaining an *R*^*2*^ of 9.3%. However, not all of the 9.3% is explained by environmental SES. Some of it is genomic. The 9.3% excludes the effect of offspring genome, but includes about one half of the effect of the genomic component in parental SES.

To weigh the relative contribution of offspring genome vs. that of SES, we’d like to directly compare the incremental *R*^*2*^ of offspring genome with that of the environmental component in parental SES (Fig. 1). But the latter is unknown even though we know that it must be smaller than 9.3% because the 9.3% results from the impact of environmental parental SES and the impact of one half of the genomic component in parental SES. Drawing from the findings of Kong et al.^16^ a rough upper bound may be set at 1.5% for the incremental *R*^*2*^ of the genomic component in parental SES. Kong et al.^16^ estimates the effect of transmitted parental alleles to be three times as large as that of non-transmitted parental alleles. This suggests that the effect of the genomic component in parental SES is about two thirds of the effect of offspring genome; the genomic component is subject to the influence of non-transmitted alleles as well as transmitted alleles. This result suggests that the genomic component in parental SES has an effect smaller than that of offspring genome. Assuming that the genomic component in parental SES has an impact equal or smaller than that of offspring genome (which has an incremental *R*^*2*^ of 1.5%), we set the upper bound of the incremental *R*^*2*^ of the genomic component in parental SES to 1.5%. Thus, our rough estimates of the incremental *R*^*2*^s for offspring genome, the genomic component in parental SES, and the environmental component in parental SES are 1.5%, 1.5% and 9.3 − 1.5 = 7.8%, respectively.

We performed additional analyses and present the results in Supplementary Materials to demonstrate the robustness of the effect of offspring genome. The sibling-fixed-effects model adds evidence that offspring genome indeed predicts verbal ability net of parental genomes in spite of a much smaller sample (Supplementary Table 1). The fixed-effects model may be an over-stringent test in this context. It controls for the genomic component in parental SES and it also unduly controls for some of the effect of offspring genome.

Replacing multiple SES measures by a single summary score of SES produces a set of findings (Supplementary Tables 2–5) that are parallel to those in Tables 2–5. The coefficients of the SES summary score are always highly significant and in the same direction as the SES measures, telling a substantially similar story as those in Tables 2–5. The two approaches each have advantages. The SES summary score is much simpler and the simplicity in the measurement of SES is almost a necessity when carrying out a G×E-interaction analysis. On the other hand, specific SES measures are directly from survey items and can be interpreted much more intuitively.

When general health, mental health and delinquency are entered into the model with SES and the two PGSs, only mental health negatively predicts verbal ability. Importantly, these additions do not change the findings from a model with parental SES and the two PGSs presented earlier.

To further test what SES predictors would remain important to the Wave-3 verbal ability, above and beyond what had already gone into the Wave-1 ability, we condition the prediction of the Wave-3 ability on the Wave-1 ability (Table 5). A coefficient of about 0.5 of Wave-1 ability indicates that the Wave-1 score predicts the Wave-3 score with about 50% accuracy. Conditional on the Wave-1 ability, the effects of the PGS and SES predictors carry a distinct meaning. These effects represent effects additional to measured and unmeasured PGSs, SES and other factors that had already acted upon the Wave-1 ability. In the findings, two SES predictors stand out: number of years of schooling by Wave 3 and neighborhood disadvantage at Wave 1. The large effect of year of schooling by Wave 3 is particularly noteworthy. Years of schooling is not used in analysis presented in Tables 2–4 because the study participants at Wave 1 were aged 12–18 with little variation in schooling after controlling for age. The coefficient of 0.78 implies that an additional year of schooling is associated with about 0.8 point of verbal ability. Four measures are taken to address the difficulty of estimating the causal effect of schooling. First, the ability PGSs are included. Second, the model controls for an earlier version of verbal ability. These controls are equivalent to controlling for the ability for seeking and attaining education. Third, the model controls for age at which the Wave-3 test is taken because of its correlation with schooling. Lastly, we measure schooling by the number of years of schooling before verbal ability is taken at Wave 3. The above comments for schooling could be also said about neighborhood disadvantage at Wave 1, which adds prediction to verbal ability after the conditioning.

Our gene-environment interaction analysis shows a positive interaction between the SES summary score and the education PGS. The interpretation could be interpreted in two ways. A favorable parental SES enhances the effect of the PGS or a higher PGS augments the advantage of a favorable parental SES. G×E-interaction analysis has numerous pitfalls and obtaining credible results is challenging. Before arriving at our current G×E results, we checked against potential issues inherent in G×E-interaction analysis^49–51^. In addition, A G×E-interaction result based on a PGS assumes that the interaction is uniform over the genome. As we discussed earlier, a part of E is genetic.

In conclusion, including both SES and offspring genome in a model of verbal ability results in about a 20% reduction in the effect size of the education PGS and about 10–15% reduction in the effect sizes of SES. The latter implies that about 20–30% of the parental SES effects are not environmental, but genomic. The three incremental *R*^*2*^s corresponding to the two PGSs, the genomic component in parental SES, and the environmental component in parental SES are about 1.5%, 1.5%, and 7.8%, respectively. Thus, the total genomic *R*^*2*^ and the total environmental *R*^*2*^ are estimated to be 3% and 7.8%, respectively.

These findings have implications and challenges for work that is devoted to the study of genetic influence on cognitive ability and for work that focuses on social environmental influences. For the former, our analysis replicates previous finding that offspring genome has a robust and substantial effect on verbal ability even after stringent controls for parental genomic influences. However, the incremental *R*^*2*^ of 1.5% appears surprisingly small, especially in view of the much larger ability heritability of 50% or more from twin studies. The latest GWAS of cognitive ability report *R*^*2*^s of 4.3%^67^ and 5.2%^14^, respectively. These estimates do not exclude the effects of parental genomes and PCAs.

The work on genomic influence at DNA level on cognitive ability is ongoing. Substantially larger samples than currently used could be assembled to hunt for more genes in fresh GWAS. Technological breakthroughs in genomics happen all the time. These foreseeable and unforeseeable advances will in all likelihood raise the percentage of variance that can be explained by offspring genomic measures in the future.

The news for social scientists studying social environmental influences is two-fold. Social scientists have long considered SES context fundamentally important in shaping life outcomes including cognitive ability. With the incremental *R*^*2*^ of environmental parental SES estimated to be roughly about 7.8% vs. 1.5% for offspring genome and 1.5% for the genomic component in parental SES, the current evidence confirms the importance of SES context. After all, the development of cognitive ability including verbal ability depends heavily on the context of modern education and society, which in turn are closely related to parental resources and decisions.

The much large incremental *R*^*2*^ of environmental parental SES than that of offspring does not support the dismissal of shared environmental influences. The dismissal is based on the observation that in twin studies the variance due to shared environmental factors tends to go to zero when individuals reach maturity. The discrepancy between twin studies and our analysis may be partially explained by the hypothesis that parental SES is positively correlated with individual-level environmental influences, that is, a higher level of parental SES affords more resources for individual-level interventions. In social-science studies, most SES measures are at shared family level.

Yet, the role of inheritance cannot be ignored. Not only an individual’s genome has an important direct influence on verbal ability; parental genomes also influence verbal ability through parental SES. Our findings suggest that about 20–30% of the conventionally-estimated coefficients of SES are not environmental but parentally genomic. The decades-long blueprint in research of including SES in a model and interpreting their effects as those of SES environment needs to be amended accordingly. A straightforward and ready solution is to include both SES and offspring genome in the same model. This would result in more accurate estimates of the effects of SES and offspring genome. The genetic effects would become more truly a reflection of children’s own genome rather than his or her parents’ genomes. More importantly, about 50% of the upward bias in the effects of parental SES due to parental genomes would be corrected. These findings point to the benefits of collecting DNA data in large social-science studies like Add-Health granted that their primary purpose is to understand social and environmental influences.

## Methods

### Data source

We use data from Add Health (http://www.cpc.unc.edu/projects/addhealth/), which is an ongoing longitudinal study of a nationally representative sample of more than 20,000 adolescents in grades 7–12 or ages 12–18 in 1994–95 in the United States who have been followed for more than 25 years^15^. Add Health has conducted one in-school survey in 1994–1995, and five in-home interviews in 1994–1995 (Wave 1), 1996 (Wave 2), 2001–02 (Wave 3), 2008 (Wave 4), and 2016–8 (Wave 5). In accordance with the University of North Carolina School of Public Health Institutional Review Board guidelines which are based upon the Codes of Federal Regulations on the Protection of Human subjects 45CFR46, participants in Add Health provided written informed consent to participate in all aspects of the study. The original purpose of Add Health was to understand the causes of health, health behavior, and educational development with a special emphasis on the role of social context at the levels of family, neighborhoods, and communities.

We start with a sample of 9975 individuals for whom GWAS measures are available. Excluded are those without a measure of verbal ability, those self-identifying as African Americans, Asian Americans, Native Americans or missing on race/ethnicity, and those with covariates missing on neighborhood disadvantage. Our final analysis sample includes 7194 individuals consisting of 5820 whites and 1374 Hispanic whites.

In January 2015, Add Health completed genome-wide genotyping on the Wave IV participants who consented to archive their DNA for future studies. Of the 15,701 respondents interviewed, 12,200 of the eligible respondents agreed to archive their DNA for future analysis “related to long term health.” Add Health utilizes two Illumina platforms for GWAS: the Illumina Human Omni1-Quad BeadChip at first and then the Illumina Human Omni-2.5 Quad BeadChip. The two platforms utilize tag SNP technology to identify and include, respectively, >1.1 million and 2.5 million genetic markers, which are derived from phases 1–3 of the International HapMap Project^68^ and the 1000 Genomes Project (1KGP)^69^.

*Verbal Ability* is measured by an abridged version (PVT) of the Peabody Picture Vocabulary Test-Revised (PPVT-R) implemented twice at Waves 1 and 3 of Add Health. PVT includes 87 or half of the items of PPVT-R. Our analysis uses the standardized score of PVT as the outcome variable. PPVT was first published in 1959 and has been revised three times^70^. Psychological literature considers PPVT an estimate of verbal intelligence^71,72^. The correlations between PPVT and full-scale intelligence tests were found to be between 0.40 and 0.60^72^.

Mother’s and father’s education are, respectively, coded into a four- category categorical variable with less than high school, high school graduation and some college, at least college degree, and missing. Mother’s and father’s occupation in Add Health originally have 16 categories. They are combined into five categories of none and other; manual or blue collar; sales, service, or administrative; professional or managerial; and missing. In the G×E-interaction analysis and the analysis presented in Supplementary Materials, we construct an SES PCA, of which occupation is a component. We test the robustness of three continuous occupation scales: the original 5 categories in Add Health, the 8-level, and the 11-level scales. In the last two, the Add Health 16 categories are mapped onto a well-known occupational prestige ratings^73^. The three sets of results are almost identical. The findings from the 11-level occupation scale are presented.

Household income is total family income, which is only available from the parental questionnaire at Wave 1 in 1994. Household income is coded into a six-category variable with cutoff points at 20^th^, 40^th^, 60^th^, and 80^th^ percentile plus a category of missing. Family structure is measured by a dichotomous variable taking the value of one if the respondent lives with two biological parents and zero if the respondent is from a household of a single parent, a stepparent, an adopted family, or a foster home at Wave 1. Sibship size is the number of siblings living in the household at Wave 1.

To capture neighborhood disadvantage, we follow the approach used by Wodtke, Harding and Elwert^74^. Neighborhood disadvantage is measured by the first principal component from a principal component analysis of six neighborhood measures that include the proportion of households living below the poverty line, the proportion of adults who are unemployed, the proportion of female-headed households, the proportion of adult residents without a high school diploma, the proportion of residents with a college degree, and the proportion of workers holding managerial or professional jobs. *In school* is coded as one if the respondent is in a school session when interviewed or if the respondent is in school in the past school year when the respondent is interviewed in a summer break; and *in school* is coded as zero otherwise.

In generating covariates, we take advantage of the longitudinal design of Add Health. Whenever it is possible to match the repeated measures of verbal ability at Waves 1 and 3, we use time-varying covariates measured at Waves 1 and 3. For example, we generate two measures of neighborhood disadvantage derived from two rounds of principal component analysis using data at Waves 1 and 3, respectively. These two measures of neighborhood disadvantage are included longitudinally in our analysis. Similarly, parental education and occupation are also measured at Waves 1 and 3 and included as time-varying covariates in analysis.

The genomic measures of educational attainment and cognitive ability are based on the 2018 GWAS of years of schooling^13^ and the 2018 GWAS of intelligence^14^, respectively. In the former, the GWAS separately regresses each of a large number of single nucleotide polymorphisms (SNPs) on years of schooling where SNPs are a particular type of genetic variables taking values 0, 1, or 2. The value represents the number of risk alleles at the genetic locus. The GWAS obtained one *β* from each SNP regression.

The GWAS results are calculated into a summary polygenic score (PGS)^75^ for each individual using PLINK. To tap all the predictability of a GWAS, a PGS for education for individual *i* is calculated using all *β*s from GWAS as weights instead of excluding SNPs above a certain P value for the observed risk alleles X_*ij*_*s* for this individual *i*: $PGS_i \,=\, \mathop {\sum}\nolimits_{j \,=\, 1}^n {\beta _{ij}{{{\mathrm{X}}}}_{ij}}$, where *j* indexes SNP and *n* stands for the total number of SNPs used in the calculation. The PGS for cognitive ability is similarly constructed.

To facilitate interpretation, a *PGS* is standardized into a Z score: $PGS_i^s \,=\, \left[ {PGS_i \,-\, M} \right]/\sigma$, where M is the mean *PGS* averaged over all individuals in the sample and *σ* is the standard deviation of the *PGS*. When $PGS_i^s$ is included in a regression model predicting verbal ability, its coefficient can be interpreted as the effect of one standard deviation of the *PGS*. Thus, the standardized *PGSs* are a way to estimate and interpret the overall genomic influence on a phenotype. The standardization is performed three times separately: within European Americans, within Hispanic whites, and within the combined sample of European and Hispanic white Americans. We have similarly constructed standardized *PGS_*for cognitive ability. In the rest of this article, we use *PGS*_*i*_ to represent $PGS_i^s$ for simplicity. The original GWAS of education and cognitive ability include data from Add Health and in this project, the Add Health PGSs are calculated using the GWAS results with Add-health data excluded. For more information on the QC procedures, imputation, LD patterns and PGS construction, see Okbay et al.^76^ at https://addhealth.cpc.unc.edu/wp-content/uploads/docs/user_guides/AH_GWAS_QC.pdf.

### Analytical strategies

Appropriate statistical procedures were used to address the correlations in verbal ability measures in Add Health^77^. One source of the correlation is due to the inclusion of the two measures of verbal ability per individual. The other source of the correlation originates from the study design of Add Health, which includes a genetic-informative sample consisting of full siblings, DZ twins, MZ twins, and other related individuals. In our analysis, we address the data hierarchy using multilevel regression models^78–80^. We implement two forms of multilevel models. The first is a three-level model that addresses the repeated measures of verbal ability in addition to the sibling clusters:

1 $$\begin{array}{*{20}{l}} {Vability_{tij}}\, =\, {\beta _{0\;ij(s)} \,+\, {\boldsymbol{SES}}_{\boldsymbol{ij}}^\prime {\boldsymbol{B}}_{\boldsymbol{1}} \,+\, {\boldsymbol{G}}_{\boldsymbol{ij}}^\prime {\boldsymbol{B}}_{\boldsymbol{2}} \,+\, {\boldsymbol{C}}_{\boldsymbol{ij}}^\prime {\boldsymbol{B}}_{\boldsymbol{3}} \,+\, e_{tij}, ({{{\mathrm{level}}}}\;1\;{{{\mathrm{model}}}})}\\ {\beta _{0ij(s)}} \, =\, {\beta _{j(s)} \,+\, v_{ij}, ({{{\mathrm{level}}}}\;2\;{{{\mathrm{model}}}})} \\ {\beta _{j(s)}} \,= \, {\beta _0 \,+\, \mu _{j(s)}, ({{{\mathrm{level}}}}\;3\;{{{\mathrm{model}}}})}\end{array}$$

where *Vability* stands for verbal ability; the subscripts *t*, *i*, *j*, and *s* index Add-Health Wave, individual, sibship and type of sibship, respectively; ***SES***, ***G*** and ***C*** are, respectively, vectors of SES, *PGSs*, and other variables including demographic indicators and principal components for addressing population admixture; ***B***_***1***_, ***B***_***2***_, and ***B***_***3***_ are vectors representing the effects of these observed variables; and *e*_*tij*_, *ν*_*ij*_, and *μ*_*j*(*s*)_ are random effects at the levels of Add-Health Wave, individual and sibship, respectively. We estimated models that distinguish different types of sibship and models that do not make that distinction, which is equivalent to ignoring the subscript *s*. The two sets of estimated coefficients of observed variables are essentially identical. We only present estimates from the models that do not make the distinction.

Our model that conditions on Wave-1 verbal ability is a two-level model that uses verbal ability at Wave 3 as the dependent variable and that estimates the effects of the same set of predictors while controlling for verbal ability at Wave 1:

2 $$\begin{array}{l} {VabilityW3_{ij}} \,=\, {\beta _{0\;j(s)} \,+\, {\boldsymbol{\alpha}} VabilityW1_{ij} + {{{\boldsymbol{SES}}}}_{{{{\boldsymbol{ij}}}}}^\prime {{{\boldsymbol{B}}}}_1 \,+\, {{{\boldsymbol{G}}}}_{{{{\boldsymbol{ij}}}}}^\prime {{{\boldsymbol{B}}}}_2 \,+\, {{{\boldsymbol{C}}}}_{{{{\boldsymbol{ij}}}}}^\prime {{{\boldsymbol{B}}}}_3 \,+\, e_{ij},\left( {{{{\mathrm{level}}}}\;1\;{{{\mathrm{model}}}}} \right)} \\ {\beta _{0j\left( s \right)}} \,=\, {\beta _0 \,+\, \mu _{j(s)}, \left( {{{{\mathrm{level}}}}\,2\,{{{\mathrm{model}}}}} \right)} \hfill \end{array}$$

Adding Wave-1 verbal ability allows a further test of the effects of offspring genome and family-SES in addition to all genomic and environmental influences that had already acted on Wave-1 verbal ability. Both (1) and (2) are random-intercept models.

The regression models are fit by the *mixed* command in STATA 16 SE. Population admixture or population stratification is a major concern in genetic association studies. Population groups separated over the past 50,000–100,000 years are likely to have developed private genetic variants that differ across population groups and that are unrelated to cognitive ability. If these population groups differ in ability test scores for environmental reasons and if these private variants are not controlled properly, the related genetic variants could be erroneously interpreted as causing differences in cognitive ability. The error can be avoided by the common practice of including the ten or so largest principle components (PCAs) in the regression that links genetic variants to a phenotype^81^. The PCAs represent ancestral genetic differences among population groups and are highly correlated with self-reported race/ethnicity. G×E-interaction terms can be added to models (1) and (2) readily. Findings are presented based on analysis in which missing values in predictors are coded into a separate category. Alternatively, missing values are addressed by performing the multiple imputation via chained equations (MICE)^82^. The *MICE* approach is accomplished by the *mim* command and the *xtmixed* command in STATA 16 SE.

### Reporting summary

Further information on research design is available in the Nature Research Reporting Summary linked to this article.

## Supplementary information

Supplementary Materials

Reporting Summary Checklist

## Data Availability

The data supporting this work are available from the Add-Health website https://addhealth.cpc.unc.edu/) but restrictions apply to the availability of the data. The restricted-use data can however be available via contractual agreement with the Carolina Population Center (CPC Data Portal: https://data.cpc.unc.edu/projects/2/view).
