# Supplementary material for: Socioeconomic and genomic roots of verbal ability from current evidence
Source: NPJ Sci Learn. 2022 Sep 9;7:22. doi: 10.1038/s41539-022-00137-8 (PMC9463438; doi:10.1038/s41539-022-00137-8)
Supplement: Supplementary file 1 — Supplementary Materials [file 41539_2022_137_MOESM1_ESM.pdf]

## SUPPLEMENTARY MATERIALS

### Supplementary Notes

In this section, we define and discuss some of the genetics terms and concepts that appear in the article.

For decades before DNA data were widely available, analysis of siblings and twins were the primary approach for attaining genetic evidence. Such analysis requires clusters of individuals with known genetic relatedness such as DZ twins, who share 50% of genetic variants on average, and MZ twins, who share 100%. Such analysis yields an estimate of **heritability ($h^2$)** of a sample or the proportion of variance in a trait due to genetic influence. Under a number of assumptions, heritability can be obtained by twice the difference between the MZ correlation and the DZ correlation or $h^2 = 2(r_{MZ} - r_{DZ})$. This result refers to a sample or population and is obtained without DNA data. Twin and related studies made invaluable contributions to the understanding of the genetic nature of human traits. However, such analysis yields only parameters at the population level. Heritability can be calculated for a subpopulation defined by environmental variables, but these are still population parameters rather than mutually adjusted genetic and environmental effects at individual level. For estimating such effects, mutually-correlated genetic and environmental measures for each individual are indispensable.

For social scientists who just begin to understand statistical analysis of genetic data, the **human genome** in an individual may be viewed as consisting of 3 billion DNA units or chemical base pairs. Only about 0.1% of the 3 billion units differ across individuals. This small proportion still amounts to millions of differences. The objective of statistical genetic analysis is to find creditable associations between these differences across individuals and a trait.

A trait can be a disease such as type 2 diabetes or a characteristic such as hair color. A tiny portion of human traits are Mendelian. **Mendelian human traits** follow the Mendelian patterns of inheritance, first described by Gregor Mendel about 150 years ago, and are determined by one genetic locus. Examples of Mendelian traits include cystic fibrosis and color blindness. **Complex human traits** are traits that do not follow the Mendelian patterns of inheritance, and generally influenced by multiple genetic variables as well as multiple environmental variables. Examples of complex traits include body mass index, height, educational attainment, and cognitive ability. Most of human traits are complex. All social outcomes are complex.

**Association studies using candidate genes** aims at finding statistical association between genetic variation among a limited number of pre-specified genes and a trait. This approach was a main method for the study of complex traits before the era of **genome-wide association studies** (**GWAS**). The candidate-gene studies are typically based on a sample size ranging from a few dozens to a few thousands of individuals. Many results fail to replicate because of the lack of statistical power. Even when such a study is successful, its findings are limited and do not cover all variations in the entire genome for the trait.

As a reaction to the inadequacies of the candidate-gene approach, GWAS aims at finding statistical association between all possible genetic variations in a genome and a trait. In a current GWAS, the variation across the genome in the 0.1% of the 3 billion DNA units is measured by about 0.1%x3billions=3 million **single-nucleotide polymorphisms** (**SNP**). SNPs are a particular type of genetic variables that can be coded as a count of number of risk alleles ranging 0, 1 or 2 in value. To establish which of the 3-million SNPs are associated with a trait, GWAS conducts 3-million separate regression analyses one for each SNP. To overcome false positives, GWAS sets the critical value at $5x10^{-8}$ and requires a replication of the findings in an independent dataset. Note that the critical value is far more stringent than the conventional 0.05. The SNPs that are found associated with a trait under the two criteria are referred to "**top hits**." GWAS top hits tend be substantiated by subsequent studies in independent data sets.

**Polygenetic scores** (**PGSs**) or genetic risk scores are one summary measure for the genome-wide influence on a trait. A PGS can be based on the top hits in a GWAS, which is equivalent to setting $P>5x10^{-8}$ , or on all of the SNPs used in a GWAS, which is equivalent to setting P=1. As described in the section titled **Genomic measures** in our article, a PGS is a sum of a number of risk allele times the coefficient of the corresponding GWAS SNPs. For example, the total number of top-hit loci based on the latest GWAS of BMI is 97 (Locke et al. 2015) and the corresponding PGS would sum over 97 loci. When a PGS is based on $P>5x10^{-8}$, not all SNPs can be used in the construction of a PGS because of **linkage disequilibrium** (**LD**) referring to the phenomenon that SNPs in a section of genomic sequence tends to be correlated. Including all the highly correlated SNPs would exaggerate the value of a PGS since that is equal to overcounting SNP effects. The reduction of the correlation among the SNPs included in a PGS is achieved by **pruning** (which removes some of the highly correlated SNPs) and **clumping** (which keeps only one SNP in a section in which the SNPs are highly correlated).

**Supplementary tables**

Supplementary table 1. Testing the robustness of the effects of ability PGSs after controlling for observed family SES and for family fixed effects: Add Health (Full siblings and DZ twins)

| | Random Effects | Random Effects | Fixed Effects |
|---|---|---|---|
| | PGS | SES+PGS | SES+PGS |
| Predictors | β(S.E.) | β(S.E.) | β(S.E.) |
| **Ability PGSs** | | | |
| PGS for Education | 2.42(0.47)*** | 1.81(0..46)*** | 1.37(0.72)* |
| PGS for IQ | 0.10(0.45) | 0.35(0.43) | 0.66(0.71) |
| **Family SES** | Not considered | Observed SES included | Controlled as fixed effects |
| | | | |
| Number of families | 354 | 354 | 354 |
| Number of individuals | 719 | 719 | 719 |

*** p<0.001, ** p<0.01, * p<0.05, + p<0.1

Results that use the first principal component as a summary measure for specific SES predictors. In addition to the presentation of the results that use specific SES measures in the main body of the article, we present results based on a principal component analysis. In these models, the first principal component is used as a summary of SES measures including mother's education, father's education, mother's occupation, father's occupation, household income, family structure, sibship size, neighborhood disadvantage, and whether the respondent was in school in the past school year. The principal component analysis was conducted only on cases with complete information on each of the SES measures. The advantage of using a single principal component is that the analysis is much simpler. In this analysis, the summary measure is almost always highly significant. The results convey a very similar story as that told by using specific SES measures. Its drawback is that SES is now a black box whose effects cannot be easily interpreted.

Supplementary table 2. Multilevel models of Wave-1&3 verbal ability: Coefficients (standard errors) of an SES principal component among Whites, Hispanics Whites and both: Add Health

| | Whites | Hispanic Whites | Whites & Hispanic Whites |
|---|---|---|---|
| | SES | SES | SES |
| Predictors | β(S.E.) | β(S.E.) | β(S.E.) |
| **SES PC** | 3.26(0.16)*** | 3.54(0.53)*** | 3.45(0.16)*** |
| **Demographics** | | | |
| Age | 0.16(0.03)*** | 0.50(0.09)*** | 0.22(0.03)*** |
| Female | -1.27(0.31)*** | -1.15(0.99) | -1.25(0.31)*** |
| **Immigration Status** | | | |
| US Born | 1.57(0.35)*** | 2.80(1.02)** | 1.78(0.34)*** |
| Speaking English Home | 8.94(2.62)*** | 4.69(1.11)*** | 5.47(0.77)*** |
| Race and Ethnicity | | | |
| White | - | - | - |
| Hispanic | - | - | -4.10(0.52)*** |
| **Constant** | 93.0(2.68)*** | 82.7(2.15)*** | 94.6(0.97)*** |
| **Random Effects** | | | |
| $\sigma_u^2$, family-level | 1.79(0.06)*** | 2.25(0.13)*** | 1.89(0.05)*** |
| $\sigma_v^2$, person-level | 1.68(0.07)*** | 1.37(0.68)* | 1.66(0.08)*** |
| $\sigma_e^2$, wave-level | 1.99(0.01)*** | 2.47(0.03)*** | 2.11(0.01)*** |
| **Model-level Parameters** | | | |
| (-2)LogLikelihood | 55000 | 11742 | 67246 |
| Person-observations | 7,397 | 1,426 | 8,823 |
| Number of Families | 3,455 | 686 | 4,135 |
| Number of Persons | 3,831 | 735 | 4,566 |
| OLS $R^2$ | 0.10 | 0.11 | 0.16 |

*** p<0.001, ** p<0.01, * p<0.05, + p<0.1

Supplementary table 3. Multilevel models of Wave-1&3 verbal ability: Coefficients (standard errors) of ability PGSs among Whites, Hispanics Whites and both: Add Health

| | Whites | Hispanic Whites | Whites & Hispanic Whites |
|---|---|---|---|
| | PGS | PGS | PGS |
| Predictors | β(S.E.) | β(S.E.) | β(S.E.) |
| **Ability PGSs** | | | |
| PGS for Education | 2.31(0.17)*** | 2.44(0.56)*** | 2.37(0.17)*** |
| PGS for IQ | 0.27(0.17) | 0.47(0.55) | 0.34(0.17)* |
| **Population Admixture** | omitted | omitted | omitted |
| **Constant** | 102.7(0.86)*** | 101.9(1.30)*** | 102.1(0.51)*** |
| **Random Effects** | omitted | omitted | omitted |
| **Model-level Parameters** | some omitted | some omitted | some omitted |
| (-2)LogLikelihood | 55260 | 11794 | 67598 |
| Number of Persons | 3,831 | 735 | 4,566 |
| OLS $R^2$ | 0.05 | 0.07 | 0.11 |

*** p<0.001, ** p<0.01, * p<0.05, + p<0.1; “omitted” indicates that the parameters are very similar to those in previous models and omitted to avoid redundancy.

Supplementary table 4. Multilevel models of Wave-1&3 verbal ability: Coefficients (standard errors) of SES PC and ability-related PGSs among Whites, Hispanics Whites and both: Add Health

| | Whites | Hispanic Whites | Whites & Hispanic Whites |
|---|---|---|---|
| | SES+PGS | SES+PGS | SES+PGS |
| Predictors | β(S.E.) | β(S.E.) | β(S.E.) |
| **Ability PGSs** | | | |
| PGS for Education | 1.79(0.16)*** | 2.16(0.53)*** | 1.88(0.16)*** |
| PGS for IQ | 0.23(0.16) | 0.63(0.52) | 0.32(0.16)* |
| **SES PC** | 2.90(0.16)*** | 3.15(0.53)*** | 3.08(0.16)*** |
| **Demographics** | | | |
| Age | 0.16(0.03)*** | 0.49(0.09)*** | 0.21(0.03)*** |
| Female | -1.20(0.31)*** | -1.55(0.97) | -1.18(0.30)*** |
| Immigration Status | | | |
| US Born | 1.59(0.34)*** | 2.82(1.00)** | 1.80(0.33)*** |
| Speaking English Home | 9.22(2.65)*** | 3.92(1.14)*** | 5.04(0.78)*** |
| Race and Ethnicity | | | |
| White | - | - | - |
| Hispanic | - | - | -1.43(0.78)+ |
| **Population Admixture** | omitted | omitted | omitted |
| **Constant** | 91.1(2.79)*** | 86.4(2.51)*** | 94.4(1.07)*** |
| **Random Effects** | omitted | omitted | omitted |
| **Model-level Parameters** | some omitted | some omitted | some omitted |
| (-2)LogLikelihood | 54856 | 11694 | 67064 |
| Number of Persons | 3,831 | 735 | 4,566 |
| OLS $R^2$ | 0.13 | 0.15 | 0.19 |

*** p<0.001, ** p<0.01, * p<0.05, + p<0.1; “omitted” indicates that the parameters are very similar to those in previous models and omitted to avoid redundancy.

Supplementary table 5. Multilevel models of Wave-3 verbal ability: Coefficients (standard errors) of SES PC and ability-related PGSs conditional on verbal ability at Wave-1 among Whites, Hispanics Whites and both: Add Health

| | Whites | Hispanic Whites | Whites & Hispanic Whites |
|---|---|---|---|
| | SES+PGS | SES+PGS | SES+PGS |
| Predictors | β(S.E.) | β(S.E.) | β(S.E.) |
| **Verbal Ability at Wave 1** | 0.48(0.01)*** | 0.47(0.04)*** | 0.47(0.01)*** |
| **Ability PGSs** | | | |
| PGS for Education | 0.57(0.15)*** | 1.37(0.63)* | 0.73(0.17)*** |
| PGS for IQ | 0.12(0.15) | 0.16(0.61) | 0.16(0.16) |
| **SES PC** | 0.56(0.18)** | 2.09(0.69)** | 0.79(0.19)*** |
| Years of Education by Wave 3 | 0.82(0.09)*** | 0.62(0.33)+ | 0.79(0.10)*** |
| **Demographics** | | | |
| Age (Wave 3) | 0.39(0.09)*** | -0.53(0.33) | 0.25(0.09)** |
| Female | -0.35(0.29) | 0.15(1.15) | -0.21(0.31) |
| **Immigration Status** | | | |
| US Born | 0.21(0.32) | -0.55(1.18) | 0.09(0.34) |
| Speaking English Home | 2.38(2.36) | -1.64(1.35) | -0.41(0.78) |
| Race and Ethnicity | | | |
| White | - | - | - |
| Hispanic | - | - | -0.72(0.77) |
| **Population Admixture** | omitted | omitted | omitted |
| **Constant** | 33.4(3.27)*** | 58.9(8.54)*** | 39.8(2.49)*** |
| **Random Effects** | omitted | omitted | omitted |
| **Model-level Parameters** | some omitted | some omitted | some omitted |
| -2 Log-Likelihood | 25335 | 5656 | 31487 |
| Number of Persons | 3,232 | 646 | 3,872 |
| OLS $R^2$ | 0.40 | 0.28 | 0.39 |

*** p<0.001, ** p<0.01, * p<0.05, + p<0.1; “omitted” indicates that the parameters are very similar to those in previous models and omitted to avoid redundancy.

Supplementary table 6. Comparing the proportion of variance explained across models (N=7,194)

| *Table No.* | *Explanatory Variables included* | $R^2$ |
|---|---|---|
| Table 2 | Parental SES | 19.9% |
| Comparison model | Parental SES & PCAs | 20.4% |
| Table 3 | Two PGSs & PCAs | 12.6% |
| Table 4 | Parental SES & PCAs & two PGSs | 21.9% |

Testing the additional effects of general health, mental health and problem behavior. Self-reported health measures general health of the respondents at Waves 1 and 3, and is based on an answer to the question of "(I)n general, how is your health?" The answer has five categories of 1=excellent, 2=very good, 3=Good, 4=fair, and 5=Poor. To facilitate interpretation, the variable is reversely coded so that a higher value corresponds to a better outcome: 1=Poor, 2=Fair, 3=Good, 4=Very good, and 5=Excellent; and it is treated as a continuous variable in analysis.

Depression is calculated by summing the modified version of the Center for Epidemiologic Studies Depression (CES-D) scale (19 items) asked at Wave 1. The response categories for each item are: never or rarely (=0), sometimes (=1), a lot of the time (=2), and most of the time or all of the time (=3). Items 4, 8, 11, and 15 were reversely coded. See Add Health website for the list of the 19 items.

Serious delinquency is constructed from a delinquency scale using 12 questions at Waves 1 and 3. The scale is similar to those widely used in delinquency and criminal behavior research (Thornberry and Krohn 2000). The 12 questions are about physical fighting that results in injuries needing medical treatment, use of weapons to get something from someone, involvement in physical fighting between groups, shooting or stabbing someone, deliberately damaging property, pulling a knife or gun on someone, stealing amounts larger or smaller than $50, breaking and entering, and selling drugs. Respondents were asked to report how many times they had been engaged in these delinquent behaviors in the past 12 months. The answers are none, 1 or 2 times, 3 or 4 times, and 5 or more times. We recode them into 0, 1, 2, and 3, respectively, for each item. The scores are then summed up and divided it by twelve. The results are rounded up and coded into categories of none, 1 or 2 times, 3 or more times, and missing.

The findings demonstrate that when our model of parental SES and offspring genome is added with general health, mental health and delinquency, only mental health significantly predicts verbal ability with a negative impact.

Supplementary table 7. Multilevel models of Wave-1&3 verbal ability: Coefficients (standard errors) of SES Context, ability PGSs, general health, mental health and problem behavior among Whites, Hispanics Whites and both: Add Health

| | Whites | Hispanic Whites | Whites & Hispanic Whites |
|---|---|---|---|
| | SES+PGS | SES+PGS | SES+PGS |
| Predictors | β(S.E.) | β(S.E.) | β(S.E.) |
| **Ability PGSs** | | | |
| PGS for Education | 1.66(0.14)*** | 1.68(0.41)*** | 1.69(0.14)*** |
| PGS for IQ | 0.23(0.13)+ | 1.01(0.41)* | 0.39(0.13)** |
| **SES Context** | | | |
| Mother's Education | | | |
| Less than high school | - | - | - |
| High school/some college | 2.78(0.55)*** | -0.46(1.36) | 2.02(0.51)*** |
| ≥college graduation | 5.23(0.68)*** | 3.50(1.95)+ | 4.70(0.66)*** |
| Missing | -0.59(1.04) | -2.15(2.27) | -1.10(0.94) |
| Father's Education | | | |
| Less than high school | - | - | - |
| High school//some college | 1.70(0.53)** | 3.19(1.31)* | 1.87(0.50)*** |
| ≥college graduation | 1.92(0.65)** | 0.59(1.86) | 1.68(0.63)** |
| Missing | 0.90(0.93) | 0.55(2.24) | 0.54(0.87) |
| Mother's Occupation | | | |
| Manual or blue collar | - | - | - |
| None & Others | -0.22(0.38) | 0.30(0.95) | -0.15(0.36) |
| Sales/service/administrative | 0.37(0.39) | -0.27(1.17) | 0.23(0.38) |
| Professional or managerial | 0.24(0.40) | 0.44(1.26) | 0.25(0.40) |
| Missing | -0.40(0.80) | 0.26(2.04) | -0.21(0.77) |
| Father's Occupation | | | |
| Manual or blue collar | - | - | - |
| None & Others | 0.01(0.39) | -0.27(1.06) | -0.09(0.38) |
| Sales/service/administrative | 1.09(0.51)* | 1.76(1.99) | 1.22(0.53)* |
| Professional or managerial | 2.14(0.33)*** | 2.08(1.18)+ | 2.21(0.34)*** |
| Missing | 0.59(0.40) | 0.45(1.05) | 0.40(0.38) |
| Household Income Wave 1 | | | |
| 0-20 percentile | - | - | - |
| 20-40 percentile | 0.75(0.57) | 3.92(1.29)** | 1.71(0.53)** |
| 40-60 percentile | 1.74(0.55)** | 5.84(1.37)*** | 2.62(0.51)*** |
| 60-80 percentile | 2.25(0.55)*** | 4.58(1.57)** | 2.89(0.53)*** |
| 80-100 percentile | 2.70*(0.58)** | 6.26(1.86)*** | 3.37(0.56)*** |
| Missing | 0.71(0.54) | 0.33(1.17) | 0.76(0.49) |
| 2 Biological Parents Wave 1 | -0.08(0.33) | -1.15(0.92) | -0.30(0.32) |
| Sibship size | | | |
| No sibling | - | - | - |
| 1 to 2 siblings | -2.14(0.67)** | -0.91(2.07) | -1.94(0.66)** |
| 3 to 5 siblings | -2.43(0.69)*** | -2.62(2.11) | -2.46(0.68)*** |
| 6 to 20 siblings | -2.78(0.74)*** | -3.29(2.19) | -2.99(0.73)*** |
| Other and Missing | 2.68(4.10) | -3.46(9.71) | 0.90(3.85) |
| Neighborhood Disadvantages | -0.40(0.10)*** | -0.95(0.32)** | -0.58(0.10)*** |
| In School | 1.40(0.31)*** | 0.60(1.02) | 1.29(0.31)*** |
| **General Health and Health Behaviors** | | | |
| Self-reported Health | 0.10(0.12) | -0.28(0.34) | 0.04(0.12) |
| Serious Delinquency | | | |
| None | - | - | - |
| 1 or 2 times | -0.14(0.21) | 0.44(0.67) | -0.02(0.21) |
| 3 or more times | -0.99(0.88) | 3.08(2.13) | 0.02(0.83) |
| Missing | -2.11(0.80)** | -2.86(1.80) | -2.45(0.74)*** |
| Depression Wave 1 | -0.18(0.02)*** | -0.25(0.05)*** | -0.19(0.02)*** |
| **Demographics** | | | |
| Age | 0.32(0.04)*** | 0.62(0.15)*** | 0.39(0.04)*** |
| Female | -0.93(0.26)*** | -0.01(0.77) | -0.72(0.26)** |
| **Immigration Status** | | | |
| US Born | 1.42(0.28)*** | 3.24(0.77)*** | 1.74(0.27)*** |

| | | | |
|---|---|---|---|
| Speaking English at Home | 6.29(2.04)** | 3.03(0.92)** | 4.13(0.61)*** |
| Race and Ethnicity | | | |
| White | - | - | - |
| Hispanic | - | - | -1.02(0.64) |
| **Population Admixture** | omitted | omitted | omitted |
| **Constant** | 86.8(2.53)*** | 83.3(4.47)*** | 87.6(1.56)*** |
| **Random Effects** | omitted | omitted | omitted |
| **Model-level Parameters** | some omitted | some omitted | some omitted |
| (-2)LogLikelihood | 77478 | 19870 | 98172 |
| Number of Persons | 5,819 | 1,374 | 7,194 |
| OLS $R^2$ | 0.16 | 0.18 | 0.23 |

*** p<0.001, ** p<0.01, * p<0.05, + p<0.1; "omitted" indicates that the parameters are very similar to those in previous models and omitted to avoid redundancy.

## Supplementary References

Locke, A. E., B. Kahali, S. I. Berndt, and et al. 2015. "Genetic studies of body mass index yield new insights for obesity biology." *Nature* 518(7538):197-U401.

Thornberry, T. P., and M. D. Krohn. 2000. "The self-report method for measuring delinquency and crime." Pp. 33-83 in *Criminal Justice 2000*. Washington, D.C.: National Institute of Justice.
