# Supplementary material for: Socioeconomic and genomic roots of verbal ability from current evidence
Source: NPJ Sci Learn. 2022 Sep 9;7:22. doi: 10.1038/s41539-022-00137-8 (PMC9463438; doi:10.1038/s41539-022-00137-8)
Supplement: Supplementary file 2 — Reporting Summary Checklist [file 41539_2022_137_MOESM2_ESM.pdf]

nature portfolio

Guang Guo

Corresponding author(s): DBPR NPJSCILEARN-00497R

Last updated by author(s): Dec 20, 2021

Reporting Summary

Nature Portfolio wishes to improve the reproducibility of the work that we publish. This form provides structure for consistency and transparency in reporting. For further information on Nature Portfolio policies, see our Editorial Policies and the Editorial Policy Checklist.

Statistics

For all statistical analyses, confirm that the following items are present in the figure legend, table legend, main text, or Methods section.

n/a	Confirmed	
☐	☒	The exact sample size (*n*) for each experimental group/condition, given as a discrete number and unit of measurement
☐	☒	A statement on whether measurements were taken from distinct samples or whether the same sample was measured repeatedly
☐	☒	The statistical test(s) used AND whether they are one- or two-sided<br>*Only common tests should be described solely by name; describe more complex techniques in the Methods section.*
☐	☒	A description of all covariates tested
☐	☒	A description of any assumptions or corrections, such as tests of normality and adjustment for multiple comparisons
☐	☒	A full description of the statistical parameters including central tendency (e.g. means) or other basic estimates (e.g. regression coefficient) AND variation (e.g. standard deviation) or associated estimates of uncertainty (e.g. confidence intervals)
☐	☒	For null hypothesis testing, the test statistic (e.g. *F*, *t*, *r*) with confidence intervals, effect sizes, degrees of freedom and *P* value noted<br>*Give P values as exact values whenever suitable.*
☒	☐	For Bayesian analysis, information on the choice of priors and Markov chain Monte Carlo settings
☐	☒	For hierarchical and complex designs, identification of the appropriate level for tests and full reporting of outcomes
☒	☐	Estimates of effect sizes (e.g. Cohen's *d*, Pearson's *r*), indicating how they were calculated

Our web collection on statistics for biologists contains articles on many of the points above.

Software and code

Policy information about availability of computer code

Data collection	No software was used to collect the data. The authors obtained the National Longitudinal Study of Adolescent to Adult Health (Add Health) data from the UNC Carolina Population Center.
Data analysis	Data analysis was done using Stata 16 SE. Code available at https://github.com/mjlinmj/PGS_verbalability.

For manuscripts utilizing custom algorithms or software that are central to the research but not yet described in published literature, software must be made available to editors and reviewers. We strongly encourage code deposition in a community repository (e.g. GitHub). See the Nature Portfolio guidelines for submitting code & software for further information.

Data

Policy information about availability of data

All manuscripts must include a data availability statement. This statement should provide the following information, where applicable:

- Accession codes, unique identifiers, or web links for publicly available datasets
- A description of any restrictions on data availability
- For clinical datasets or third party data, please ensure that the statement adheres to our policy

The data supporting this work are available from the Add Health website (https://addhealth.cpc.unc.edu/) but restrictions apply to the availability of the data. The restricted-use data can however be available via contractual agreement with the Carolina Population Center (CPC Data Portal: https://data.cpc.unc.edu/projects/2/view).

Field-specific reporting

Please select the one below that is the best fit for your research. If you are not sure, read the appropriate sections before making your selection.

☐ Life sciences ☒ Behavioural & social sciences ☐ Ecological, evolutionary & environmental sciences

For a reference copy of the document with all sections, see nature.com/documents/nr-reporting-summary-flat.pdf

Behavioural & social sciences study design

All studies must disclose on these points even when the disclosure is negative.

Study description	This study is a statistical analysis of the National Longitudinal Study of Adolescent Health (Add Health) data.
Research sample	We use data from Add Health (http://www.cpc.unc.edu/projects/addhealth/), which is an ongoing longitudinal study of a nationally representative sample of more than 20,000 adolescents in grades 7-12 or ages 13-18 in 1994-95 in the United States who have been followed for more than 20 years.
Sampling strategy	This study analyzes an existing dataset, so no sampling is involved. The Add Health study selected 80 high schools from a sample frame of 26,666 school in Wave I. In total, 90,118 students completed a in-school questionnaire in Wave I. Adolescents in grades 7 to 12 who answered the in-school survey were sampled to participate in the in-home interview. Overall, 20,745 adolescents answered to the Wave I in-home interview. The sample included about 3,000 individuals who are identical twins, fraternal twins, full siblings, and half siblings. Also, Add Health has a multiracial and multiethnic sample. About 15,000 participants remained in the follow-up surveys. In January 2015, Add Health completed genome-wide genotyping on the Wave IV participants who consented to archive their DNA for future studies. Of the 15,701 respondents interviewed, 12,200 of the eligible respondents agreed to archive their DNA for future analysis “related to long term health.”
Data collection	Pen and paper questionnaire was used in the Wave I in-school interview. Computer-assisted Personal Interview (CAPI)/Audio Computer-Assisted Self Interview (ACASI) was used to interview the adolescents in the in-home interview. For the genetic data (GWAS), the saliva samples provided by the respondents were genotypes using two Illumina platforms: the Illumina Human Omni1-Quad BeadChip at first and the Illumina Human Omni-2.5 Quad BeadChip at a later time. The two platforms utilize tag SNP technology to identify and include, respectively, >1.1 million and 2.5 million genetic markers, which are derived from phases 1-3 of the International HapMap Project and the 1,000 Genomes Project (1KGP). The researchers of this study were blind to the study hypothesis during data collection.
Timing	Add Health has conducted one in-school survey in 1994-1995, and five in-home interviews in 1994-1995 (Wave 1), 1996 (Wave 2), 2001-02 (Wave 3), 2008 (Wave 4), and 2016-8 (Wave 5).
Data exclusions	We start with a sample of 9,975 individuals for whom GWAS measures are available. Our final analysis sample consists of 9,786 individuals. Excluded are those without a measure of verbal ability, those self-identifying as African Americans, Asian Americans, Native Americans and missing on race/ethnicity, and those covariates missing on neighborhood disadvantage, whether in school before the interview, self-reported health, binge drinking, marijuana use or smoking. Our final analysis sample includes 7,194 individuals consisting of 5,820 European Americans and 1,374 Hispanic European Americans. Alternatively, we replace the missing values via a multiple imputation procedure to test the robustness of our findings.
Non-participation	The response rate of the Wave IV sample collection when cohort members were 24-32 years old was 80.3%. 96% of respondents consented to provide saliva for DNA and 81% of those consented agreed to have the specimen archived.
Randomization	Participants were not allocated into experimental groups. This study uses statistical methods to control socioeconomic status and other covariates (e.g., sex, age, mother's and father's education, occupation, family income, lived with biological parents or not, sibship size, neighborhood disadvantage, whether the adolescent was in school or not, years of education, US born or not, speak English at home or not, race and ethnicity, and 10 principal components for population stratification).

Reporting for specific materials, systems and methods

We require information from authors about some types of materials, experimental systems and methods used in many studies. Here, indicate whether each material, system or method listed is relevant to your study. If you are not sure if a list item applies to your research, read the appropriate section before selecting a response.

Materials & experimental systems

n/a	Involved in the study	
☒	☐	Antibodies
☒	☐	Eukaryotic cell lines
☒	☐	Palaeontology and archaeology
☒	☐	Animals and other organisms
☐	☒	Human research participants
☒	☐	Clinical data
☒	☐	Dual use research of concern

Methods

n/a	Involved in the study	
☒	☐	ChIP-seq
☒	☐	Flow cytometry
☒	☐	MRI-based neuroimaging

Human research participants

Policy information about studies involving human research participants

Population characteristics	See above.
Recruitment	Information on recruitment of Add Health can be found here: https://www.cpc.unc.edu/projects/addhealth/design.
Ethics oversight	University of North Carolina School of Public Health Institutional Review Board

Note that full information on the approval of the study protocol must also be provided in the manuscript.
